# Expression of Fascin-1 and its diagnostic value in liver cancer

**DOI:** 10.1038/s41598-024-60026-5

**Published:** 2024-05-02

**Authors:** Shi-Ping Lu, Li-Jing Jiang, Yan Wang, Jun-Kang Shao, Zhi-Qun Du, Bi-Fei Huang, Chao-Qun Wang

**Affiliations:** 1 Department of Pathology, Affiliated Dongyang Hospital of Wenzhou Medical University, 60 Wu Ning Xi Road, Dongyang, Zhejiang China; 2 Department of Medical Oncology, Affiliated Dongyang Hospital of Wenzhou Medical University, Dongyang, Zhejiang China

**Keywords:** Fascin-1, Liver cancer, Hepatocellular carcinoma, Intrahepatic cholangiocarcinoma, Immunohistochemistry, Cancer, Tumour biomarkers

## Abstract

Although some studies have reported on the expression and clinical significance of Fascin-1 (FSCN1) in liver cancer, the clinical application and differential diagnosis value of FSCN1 in liver cancer are still unclear. The aim of this study was to analyze the expression level of FSCN1 protein in liver cancer tissues and explore its diagnostic and application value in differentiating between hepatocellular carcinoma (HCC) and intrahepatic cholangiocarcinoma (ICC). The immunehistochemical analysis was used to detect the expression of FSCN1 in 108 cases of HCC, 26 cases of ICC, 23 cases of liver cirrhosis, and 11 cases of normal liver tissues. The differences in the positive expression rate and strong positive expression rate of FSCN1 among different groups were analyzed. The positive rate of FSCN1 in normal liver tissues, liver cirrhosis, HCC, and ICC tissues was 0.0% (0/11), 0.0% (0/23), 13.9% (15/108), and 92.3% (24/26), respectively, while the strong positive rate was 0.0% (0/11), 0.0% (0/23), 0.9% (1/108), and 69.2% (18/26), respectively. Both the positive rate and strong positive rate of FSCN1 in ICC tissues were significantly higher than those in HCC, liver cirrhosis, and normal liver tissues. Additionally, the positive rate of FSCN1 in moderately to poorly differentiated HCC tissues was 18.8% (15/80), significantly higher than in well-differentiated HCC (0.0%, 0/28) (*P* = 0.031). In liver cancer, the sensitivity, specificity, positive predictive value (PPV), and negative predictive value (NPV) of FSCN1 positive prediction for ICC were 92.3%, 86.1%, 61.5%, and 97.9%, respectively, whereas the sensitivity, specificity, PPV, and NPV of FSCN1 strong positive prediction for ICC were 69.2%, 99.1%, 94.7%, and 93.0%, respectively. These results suggest that FSCN1 may play an important role in the occurrence and progression of liver cancer, and it can be used as a novel diagnostic marker for ICC.

## Introduction

Liver cancer is one of the leading causes of cancer-related deaths worldwide, with hepatocellular carcinoma (HCC) and intrahepatic cholangiocarcinoma (ICC) being the most common types^1,2^. The prevalence of liver cancer presents a substantial threat to the health and well-being of individuals worldwide. In recent years, although there have been significant advancements in the comprehensive treatment of liver cancer, the high recurrence rate after surgery and the fact that most patients are diagnosed in the intermediate or advanced stages result in poor overall treatment outcomes and high mortality rates^3–6^. Therefore, further exploration of the molecular mechanisms underlying the occurrence and progression of liver cancer is of great significance. Additionally, distinguishing between HCC and ICC is crucial for determining appropriate treatment strategies and predicting patient prognosis. However, accurately differentiating between HCC and ICC poses a challenge for pathologists, especially in cases of poorly differentiated tumors or when only liver biopsy samples are available.

Fascin-1 (FSCN1) is an actin-bundling protein that plays a key role in cell migration and metastasis^7^. Previous studies have reported on the expression and clinical significance of FSCN1 in liver cancer. Research indicates that FSCN1 may serve as a novel biomarker for the progression of HCC and an important indicator of poor prognosis in HCC patients^8,9^, and FSCN1 plays a crucial role in the formation and malignant transformation process of HCC^10^. Another study involving immunehistochemical and molecular analysis showed that overexpression of FSCN1 in ICC is associated with carcinogenesis and poor prognosis^11^. However, existing studies have not clarified whether there are differences in the expression of FSCN1 in different histological types of liver cancer, as well as its relationship with clinicopathologic characteristics such as clinical staging, histological differentiation, vascular invasion, presence of hepatitis B and cirrhosis. Therefore, the clinical application and diagnostic value of FSCN1 in liver cancer have not been fully understood. This study aimed to further investigate the expression and differences of FSCN1 in different histological types of liver cancer tissues, liver cirrhosis tissues, and normal liver tissues, and to analyze the relationship between FSCN1 expression and various clinicopathologic characteristics in patients with HCC. The goal of this study was to explore the diagnostic and application value of FSCN1 in liver cancer.

## Materials and methods

### Patients and tissue samples

A total of 134 non-consecutive liver cancer paraffin specimens (including 108 cases of HCC and 26 cases of ICC) archived in the pathology department of Affiliated Dongyang Hospital of Wenzhou Medical University (Dongyang, Zhejiang, China) between January 2008 and December 2018 were collected. Additionally, 23 paracancerous liver cirrhosis paraffin tissues and 11 normal liver paraffin tissues were collected. The inclusion criteria for cases were patients admitted and operated at the hospital, confirmed pathologically to have liver cancer or liver cirrhosis. The exclusion criteria were patients who received anti-tumor treatments such as intervention, targeted therapy, chemotherapy, radiotherapy, or immunotherapy before surgery. The pathological grading of liver cancer was conducted according to the WHO criteria (5th edition, 2019). The staging of liver cancer patients was based on the 8th edition of the American Joint Committee on Cancer (AJCC) Cancer Staging Manual. Among HCC patients, there were 91 males and 17 females with ages ranging from 34 to 79 years and a median age of 57 years. Histological differentiation revealed 28 cases of high differentiation, 69 cases of moderate differentiation, and 11 cases of low differentiation. TNM staging showed 78 cases in stage I, 9 cases in stage II, 20 cases in stage III, and 1 case in stage IV. For further details on other characteristics and groups, please refer to Table 3. As for ICC patients, there were 13 males and 13 females, with ages ranging from 40 to 76 years and a median age of 63 years. Histological differentiation revealed 1 cases of high differentiation, 22 cases of moderate differentiation, and 3 cases of low differentiation. TNM staging showed 15 cases in stage I, 2 case in stage II, 9 cases in stage III, and 0 cases in stage IV. The study received approval from the Ethics Committee of the Affiliated Dongyang Hospital of Wenzhou Medical University (2023-YX-345). Prior to participation, all participants provided written informed consent. The study methodology adhered to the applicable guidelines and regulations set forth by the Affiliated Dongyang Hospital of Wenzhou Medical University.

### Tissue array preparation and IHC analysis

Tissue Array Preparation: We followed the methods described by Wang et al.^12^. To summarize, the Quick-Ray® UT-06 tissue microarray system and the Quick-Ray premade recipient block (UB-06) wax model, both produced by Unitma Co., Ltd. in Seoul, Korea, were utilized for the preparation of tissue specimens measuring 1 mm in diameter. Two specific locations were chosen from each sample of liver cancer tissue for sampling purposes. IHC Analysis: The Envision System (Dako, Glostrup, Denmark) was used for IHC staining of paraffin-embedded tissue sections, following the method previously described^13,14^. Primary antibodies used in the experiment included the anti-FSCN1 mouse monoclonal antibody (clone 55 k-2; diluted to a concentration of 1:100; obtained from Santa Cruz Biotechnology, Santa Cruz, CA, USA). For the secondary antibody, Dako's HRP rabbit/mouse universal antibody (from Dako, Glostrup, Denmark) was employed. In the negative control, the vehicle was initially incubated, followed by the secondary antibody, without any primary antibody. As positive control, we used the internal control in the liver tissue, to check for FSCN1 staining in the interstitial fibers and vascular endothelial cells.

### Assessment of staining

The intensity and extent of FSCN1 expression were assessed in liver cancer cells, liver cells from liver cirrhosis, and normal liver tissue, using the same scoring criteria as described previously^13^. To summarize, the intensity of staining was assessed using scores of 0 (negative), 1 (weak), 2 (medium), or 3 (strong). The extent of staining was evaluated with scores of 0 (0%), 1 (1–25%), 2 (26–50%), 3 (51–75%), or 4 (76–100%). Samples with a combined score of intensity and extent greater than or equal to 3, and a percentage of cells with clear cytoplasmic staining exceeding 5%, were considered positive for FSCN1. Samples with a combined score of 6 or higher and a staining intensity of 3 were classified as strongly positive for FSCN1. Two pathologists independently scanned and scored each entire section.

### Statistical analysis

SPSS version 19.0 (SPSS Inc, Chicago, IL, USA) was used for all statistical analyses. Pearson’s chi-square test was used to compare differences/correlations between groups for qualitative variables. To evaluate the predictive ability of FSCN1 for ICC, the sensitivity, specificity, positive predictive value (PPV), and negative predictive value (NPV) of its expression were calculated. Statistical significance was defined as *P* values < 0.05.

## Results

### FSCN1 expression in liver tissues

**Figure 1:**
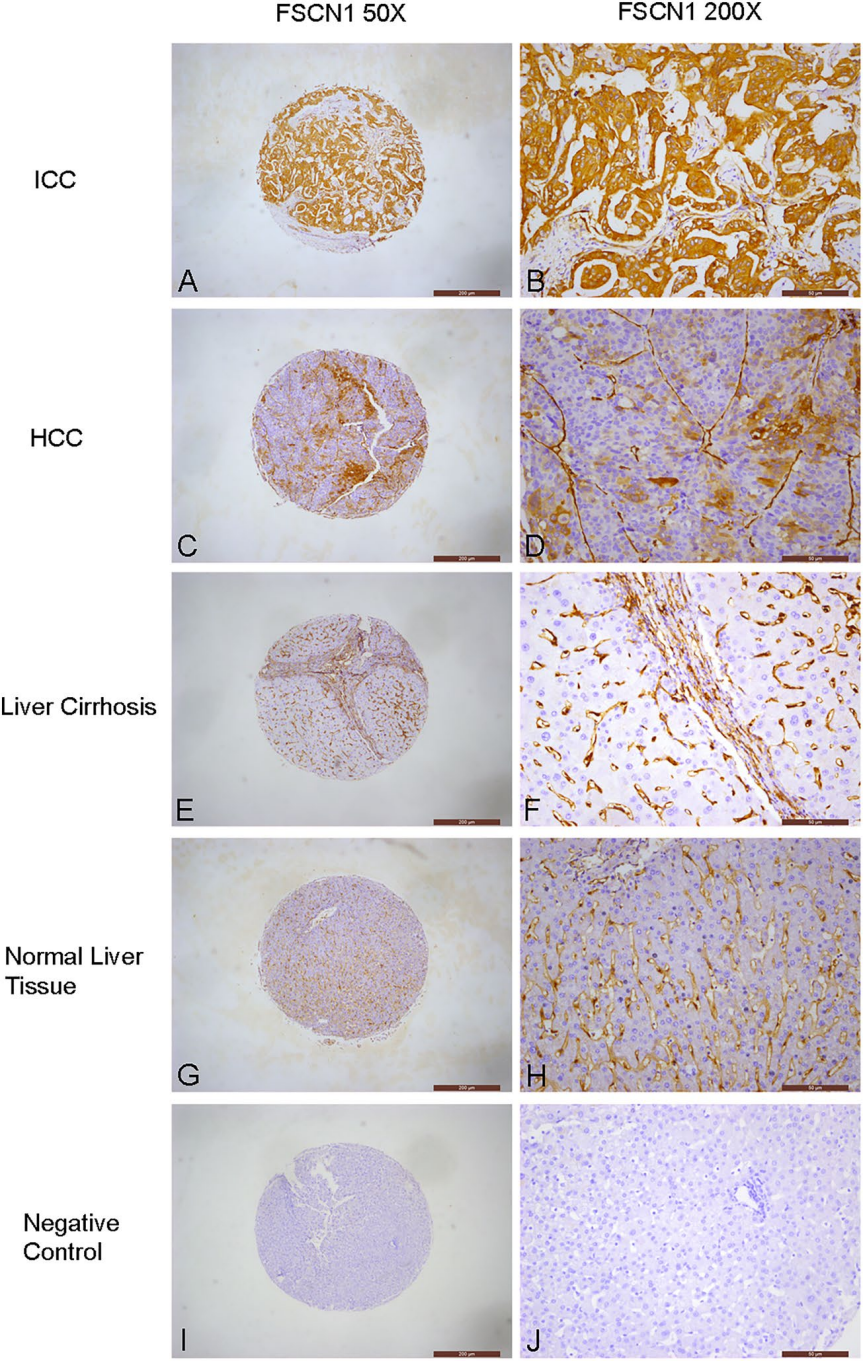
Immunochemical analysis of FSCN1 expression in liver tissues. (**A**, **B**) ICC, strongly positive fascin-1 expression in cancer cells. (**C**, **D**) HCC, positive fascin-1 expression in cancer cells. (**E**, **F**) Liver Cirrhosis, negative fascin-1 expression in liver cells. (**G**, **H**) Normal liver tissue, negative fascin-1 expression in liver cells. (**I**, **J**) Negative control, negative fascin-1 expression in liver cells, interstitial fibers and vascular endothelial cells.

Table 1 present the positivity rates of FSCN1 in various types of liver tissues, including normal liver tissues, liver cirrhosis, HCC, and ICC tissues. The rates were 0.0% (0/11), 0.0% (0/23), 13.9% (15/108), and 92.3% (24/26) respectively. Strong positivity rates were also recorded, with rates of 0.0% (0/11), 0.0% (0/23), 0.9% (1/108), and 69.2% (18/26) respectively (Table 2, Fig. 1). Notably, the positivity rates and strong positivity rates of FSCN1 in ICC tissues were significantly higher than those in HCC, liver cirrhosis, and normal liver tissues (*P* < 0.01 for all comparisons).

**Table 1:** Positive FSCN1 expression in liver tissues.

Group	No	Positive FSCN1 expression	
		Negative, n (%)	Positive, n (%)
Normal liver tissue	11	11 (100.0%)	0 (0.0%)
Liver cirrhosis	23	23 (100.0%)	0 (0.0%)
HCC	108	93 (86.1%)	15 (13.9%)
ICC	26	2 (7.7%)	24 (92.3%)

**Table 2:** Strong positive FSCN1 expression in liver tissues.

Group	No	Strong positive FSCN1 expression	
		Negative and weakly positive, n (%)	Strong positive, n (%)
Normal liver tissue	11	11 (100.0%)	0 (0.0%)
Liver cirrhosis	23	23 (100.0%)	0 (0.0%)
HCC	108	107 (99.1%)	1 (0.9%)
ICC	26	8 (30.8%)	18 (69.2%)

### Relationship between FSCN1 and clinicopathological features of HCC

Clinicopathological characteristics were collected from 108 HCC patients, including gender, age, tumor size, number of masses, tumor differentiation, vascular invasion, peritoneal invasion, T stage, tumor stage, presence of concurrent hepatitis B or cirrhosis, AFP levels, smoking history, alcohol consumption, and BMI. However, data on AFP were missing for 2 cases.

Table 3 shows that the positivity rate of FSCN1 in moderately to poorly differentiated HCC was 18.8% (15/80), which was significantly higher than the high differentiation group (0%, 0/28) (*P* = 0.031). The positivity rate of FSCN1 protein in HCC with peritoneal invasion was 31.2% (5/16), higher than the group without peritoneal invasion (10.9%, 10/92), but the difference was not statistically significant (*P* = 0.074). The positivity rate of FSCN1 protein in tumor stage II–IV group (23.3%, 7/30) was higher than in stage I patients (10.2%, 8/78), but the difference was also not statistically significant (*P* = 0.147).

**Table 3:** Association of FSCN1 expression with clinic-pathological parameters in HCC patients.

Variables	No. of patients	FSCN1 negative, n (%)	FSCN1 positive, n (%)	*P*-value
Gender				
Male	91	78 (85.7)	13 (14.3)	1.000
Female	17	15 (88.2)	2 (11.8)	
Age (years)				
≤ 60	67	59 (88.0)	8 (12.0)	0.454
> 60	41	34 (82.9)	7 (17.1)	
Tumor size (cm)				
≤ 5	84	72 (85.7)	12 (14.3)	1.000
> 5	24	21 (87.5)	3 (12.5)	
Number of masses				
Individual	100	85 (85.0)	15 (15.0)	0.516
Multiple	8	8 (100.0)	0 (0.0)	
Tumor differentiation				
High	28	28 (100.0)	0 (0.0)	**0.031**
Moderate-Poor	80	65 (81.2)	15 (18.8)	
Vascular invasion				
Negative	98	85 (86.7)	13 (13.3)	0.915
Positive	10	8 (80.0)	2 (20.0)	
Peritoneal invasion				
Negative	92	82 (89.1)	10 (10.9)	0.074
Positive	16	11 (68.8)	5 (31.2)	
T stage				
T1	79	70 (88.6)	9 (11.4)	0.355
T2–T4	29	23 (79.3)	6 (20.7)	
Tumor stage				
I	78	70 (89.8)	8 (10.2)	0.147
II–IV	30	23 (76.7)	7 (23.3)	
Hepatitis B				
No	11	10 (90.9)	1 (9.1)	0.980
Yes	97	83 (85.6)	14 (14.4)	
Liver cirrhosis				
No	37	32 (86.5)	5 (13.5)	0.935
Yes	71	61 (85.9)	10 (14.1)	
AFP				
< 400	79	66 (83.5)	13 (16.5)	0.398
≥ 400	27	25 (96.2)	2 (7.4)	
Smoke				
No	50	45 (90.0)	5 (10.0)	0.278
Yes	58	48 (82.8)	10 (17.2)	
Drink				
No	62	56 (90.3)	6 (9.7)	0.142
Yes	46	37 (80.4)	9 (19.6)	
BMI				
≤ 23.9	66	57 (86.4)	9 (13.6)	0.924
> 23.9	42	36 (85.9)	6 (14.1)	

Significant values are in bold.

### The value of FSCN1 in the differential diagnosis of liver cancer

**Figure 2:**
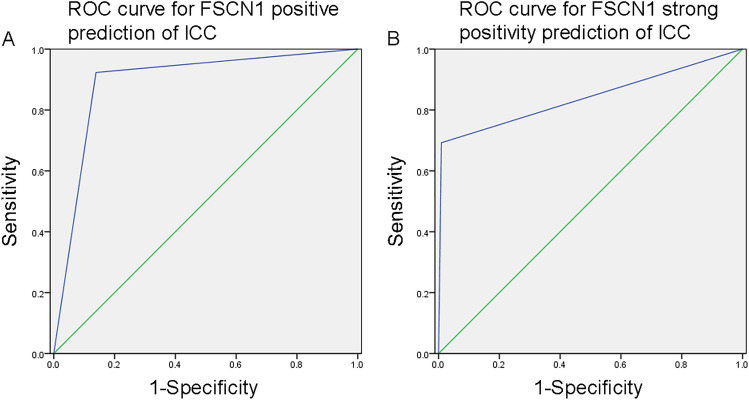
(**A**) ROC curve for FSCN1 positive prediction of ICC. (**B**) ROC curve for FSCN1 strong positivity prediction of ICC.

Due to the significant overexpression of FSCN1 in ICC, we further analyzed the value of FSCN1 in differentiating between HCC and ICC. The ROC curve analysis showed that the area under the curve (AUC) for FSCN1 positive prediction of ICC was 0.892 (95% CI 0.821–0.963; *P* < 0.01; Fig. 2A), with a sensitivity of 92.3%, specificity of 86.1%, PPV of 61.5%, and NPV of 97.9%. On the other hand, the AUC for FSCN1 strong positivity prediction of ICC was 0.842 (95% CI 0.732–0.951; *P* < 0.01; Fig. 2B), with a sensitivity of 69.2%, specificity of 99.1%, PPV of 94.7%, and NPV of 93.0%.

## Discussion

FSCN1, a cytoskeletal protein, has a molecular weight of 55 kDa and is primarily found in normal mesenchymal cells, endothelial cells, dendritic cells, and neuronal cells. Its role involves enhancing the creation of cell membrane filopodia, lamellipodia, and microspikes, ultimately resulting in heightened cell motility, migration, and the invasion and metastasis of tumor cells^7^. Multiple studies have demonstrated that FSCN1 experiences an increase in expression in different categories of tumors including breast cancer^14,15^, non-small cell lung cancer^16–18^, and gastric cancer^19,20^. This elevation substantially boosts the invasive and migratory capacities of tumor cells. Consequently, the presence of Fascin-1 occupies a crucial position in the origin and progression of tumors. Currently, a few studies have reported on the expression of FSCN1 in liver cancer, showing that FSCN1 is highly expressed in both HCC and ICC, and is associated with poor patient prognosis^8–11^. Despite this, it remains uncertain whether there are variances in the manifestation of FSCN1 among various histological types of liver cancer, such as HCC and ICC, as well as its correlation with different clinicopathological characteristics of patients. Therefore, the clinical application and diagnostic value of FSCN1 in liver cancer have not been fully understood.

This study selected 108 cases of HCC, 26 cases of ICC, 23 cases of liver cirrhosis, and 11 cases of normal liver tissue to elucidate the expression of FSCN1 in liver tissue and its clinicopathological significance in the occurrence and development of liver cancer. The results of this study showed that the positivity rate of FSCN1 in HCC was 13.9%, while the positivity rate in normal liver tissue and liver cirrhosis tissue was 0%, suggesting that FSCN1 plays an important role in the occurrence and development of HCC. Furthermore, FSCN1 expression was significantly higher in moderately to poorly differentiated HCC compared to well-differentiated HCC. Additionally, there was an increasing trend of FSCN1 expression in HCC patients with positive peritoneal invasion and higher clinical stages. These results suggest that FSCN1 is closely related to the invasiveness and metastasis of HCC and its high expression may indicate poor prognosis.

Currently, there are certain challenges in accurately distinguishing between HCC and ICC in clinical practice, especially in the case of liver biopsy specimens or poorly differentiated liver tumors, where pathologists need to use immunehistochemical staining in addition to HE staining for differentiation. Our results showed a high positivity rate of FSCN1 in ICC, reaching 92.3%, with a strong positivity rate of 69.2%. In contrast, the positivity rate of FSCN1 in HCC was only 13.9%, with a strong positivity rate of 0.9%. This indicates that the expression of FSCN1 is significantly higher in ICC compared to HCC. Therefore, we further analyzed the value of two scoring criteria, FSCN1 positivity and strong positivity, in distinguishing between HCC and ICC. The results showed that using FSCN1 positivity as a predictor for ICC had a sensitivity, specificity, PPV, and NPV of 92.3%, 86.1%, 61.5%, and 97.9%, respectively. However, when using FSCN1 strong positivity as a predictor, although the sensitivity decreased to 69.2%, the specificity and PPV were significantly increased to 99.1% and 94.7%, respectively. Therefore, we believe that FSCN1 has good discriminative value for HCC and ICC, especially when considering strong positivity as a potential biomarker for differentiating between them.

These findings suggest that FSCN1 may have an important role in the development and progression of liver cancer. Moreover, FSCN1 could serve as a novel diagnostic marker for distinguishing ICC from HCC, with high sensitivity and specificity. Further investigation of FSCN1’s clinical implications in liver cancer and its potential as a therapeutic target is warranted.

## Data Availability

All the datasets used and/or analyzed in the study are available from the corresponding author on reasonable request.
